# 

**Published:** 1889-07-13

**Authors:** 


No. 146, Vol. VI.—July 13th, 1889.

[Registered at the General Post Office as a Newspaper.]

Per Annum, 6s. 6d., post free.
Monthly Parts, 6d.; post free 7½d.
Ditto per Annum, 7s. 6d., post free.

